# Influence of operating parameters of a settling-based perfusion process on expansion of VERO cells attached on microcarriers

**DOI:** 10.1186/1753-6561-5-S8-P60

**Published:** 2011-11-22

**Authors:** Amal El Wajgali, Frantz Fournier, Eric Olmos, Cécile Gény, Hervé Pinton, Annie Marc

**Affiliations:** 1 Laboratoire Réactions et Génie des Procédés, UPR-CNRS 3349, Vandoeuvre-lès-Nancy, France; 2 Sanofi Pasteur, Marcy L’Etoile, France

## Background

The growing demand for biologicals produced by animal cells motivates the development of more efficient and reliable culture production processes. In the particular case of the industrial production of viral vaccines by Vero cells adhered on microcarriers, the cell propagation phases are mainly devoted to reach high cell density in less time while maintaining a good cell physiological state. Several papers have reported the optimization of culture conditions for microcarrier cultures [1-4]. Otherwise, perfusion bioreactors, based on continuous medium renewal and cell retention, can be a good alternative to batch systems. Among the various technologies for cell retention, gravitational settler is a promising device for large-scale perfusion culture process. This simple device takes advantage of the difference between the cell settling rate and the medium harvesting flow rate. Moreover, in the particular case of cells attached on microcarriers, it is more suitable than in the case of single suspended cells. So, the aim of this work was to evaluate the performances of adherent Vero cell cultures performed inside a perfused bioreactor using a gravitational settler as cell retention device. The study focused on the influence of two operating parameters, such as microcarrier concentration (MCs) and initial cell density (C_0_), on the cell growth.

## Materials and methods

Vero cells were provided by Sanofi Pasteur and cultivated in a serum-free medium attached on Cytodex-1 microcarriers (GE Healthcare). Cultures were performed in a 2 L bioreactor (Pierre Guérin, France) controlled at pH: 7.2, temperature: 37°C, pO_2_: 25 % with an agitation rate of 90 rpm. After 48 h of batch culture, the perfusion medium flow rate was started at 0.5 vol.d^-1^. The harvest flow rate was made through a settling glass tube. The concentration of adherent cells was measured by the crystal violet method.

A Design of Experiment (DoE) was set up, with the objective to study the effect of two operating parameters (MCs and C_0_) in order to reach high maximal cell density and rapid cell growth. The chosen criteria for DoE response were the maximal cell concentration, either per medium volume (cells.mL^-1^) or per microcarrier (cells.MCs^-1^), and the population doubling level (PDL). A D-optimal design was chosen because of the irregularity of the experimental region. Three levels were defined for each parameter. The Modde 7 software was used to generate the DoE: 7 experiments plus one repetition in order to assess the repeatability. Theoretical initial cell densities (C_0_) were corrected by the experimental values. The table 1 gives the corresponding operating parameters of the various perfused cultures.

**Table 1:** Operating conditions and experimental results for perfused experiments.

Experiments	Operating factors		Experimental results		
	
	MCs (g.L^-1^)	C_0_ (cells.cm^-2^)	C_max_ (10^6^cells .mL^-1^)	C_max_ (cells.MC^-1^)	PDL
1	2.5	9100	1.9	114	4.2
2	5	23000	3.9	116	2.9
3	1.2	6000	2.5	311	6.3
4	3.8	10100	2.7	106	3.9
5	1.2	28500	2.2	275	3.8
6	3.8	36000	3.2	125	2.4
7	2.5	14200	2.5	149	3.7
8 (7bis)	2.5	14200	2.5	147	4.2

## Results

The settling tube used as cell retention device was observed to be not only easy-to-implement but also a reliable system for retention of cells adhered on microcarriers. Indeed, no microcarrier was found in the harvest flow rate, whatever the culture operating conditions. The evolution of cell density was studied for more than 10 days. A final stabilization of the maximal cell density was observed during several days for all experiments (Table 1). The repeatability was evaluated with experiments 7 and 8, performed with the same operating parameters (MCs: 2.5 g.L^-1^ and C_0_: 14 200 cells.cm^-2^). Both experiments displayed similar cell kinetics and reached the same maximal cell density (2.5 x 10^6^ cells.mL^-1^).

On the one hand, the experimental kinetics results were compared on the basis of the maximal cell concentrations. The highest cell density per medium volume (3.9 x 10^6^ C.mL^-1^) was reached for the culture performed with the highest MCs (5 g.L^-1^, exp. 2). But the highest cell density per microcarrier (300 cells.MCs^-1^) was obtained with the lowest MCs of 1.2 g.L^-1^ (exp. 3 and 5) whatever the C_0_ value (6 000 and 23 500 cells.cm^-2^). It was also pointed out that, according to the microcarrier concentration used, the maximal cell density (in cells per medium volume) could depend on the initial cell-to-bead-ratio. Indeed, the two experiments performed with 2.5 g.L^-1^ MCs reached 1.9 x 10^6^ cells.mL^-1^ (exp. 1) when C_0_ was 9 100 cells.cm^-2^, and 2.5 x 10^6^ cells.mL^-1^ with C_0_ of 14 200 cells.cm^-2^ (exp. 7). The same conclusion was obtained with 3.8 g.L^-1^ MCs (exp. 4 and 6). Therefore, the highest cell concentration was reached when a sufficient number of cells per microcarrier was respected at the beginning of culture. But, with 1.2 g.L^-1^ MCs the maximal cell density was similar for the two C_0_ used, suggesting that the maximal cell concentration was less dependent on initial cell density at very low carrier concentration.

Furthermore, the statistical analysis of the influence of the operating parameters, on the basis of the three chosen criteria, showed that the best model was observed with the PDL values. The model predicted correctly experimental results and was described by a first-order polynomial. The regression showed satisfactory statistical qualities and neither interaction nor quadratic effects were found significant. The model indicated that low values for both operating factors MCs and C_0_ favoured a higher PDL.

## Conclusion

The easy-to-implement settling tube was a reliable system for retention of cells adhered on microcarriers. The influence of two operating parameters (MCs and C_0_) on the Vero cell growth was quantified according to three criteria related to cell growth (cells.mL^-1^, cells.MCs^-1^ and PDL). While the highest microcarrier concentration led to the highest total amount of attached cells, the lowest MCs induced the best carrier recovery by the cells. Moreover both low MCs and C_0_ values favored a high PDL. These results provide a preliminary screening of operating conditions before undertaking a rational scale-up of the perfusion process.
